# 

**Published:** 1908-10

**Authors:** 


### CONTENTS—OCTOBER, 1908.—VOL. XXIV, NO. 4.

SELECTED—

The Auto-Protective Resources of the Body—A New Foundation for Scientific Therapeutics. By Charles E. De M. Sajous, M. D., Philadelphia ........ 137

ORIGINAL ARTICLES—

Some Reflections on Psychotherapy. By Dr. Julius Grinker, Chicago ........ 159

Pyorrhea Alveolaris. By Julian Smith, D. D. S., Austin, Texas. 166

EDITORIALETS ........ 171

ABSTRACTS AND SELECTIONS ........ 176

